# Suppression of lusitropy as a disease mechanism in cardiomyopathies

**DOI:** 10.3389/fcvm.2022.1080965

**Published:** 2023-01-09

**Authors:** Steven Marston, Jose Renato Pinto

**Affiliations:** ^1^National Heart and Lung Institute, Imperial College London, London, United Kingdom; ^2^Department of Biomedical Sciences, Florida State University College of Medicine, Tallahassee, FL, United States

**Keywords:** adrenergic stimulation, lusitropy, contractility, protein kinase A, hypertrophic cardiomyopathy, dilated cardiomyopathy

## Abstract

In cardiac muscle the action of adrenaline on β1 receptors of heart muscle cells is essential to adjust cardiac output to the body’s needs. Adrenergic activation leads to enhanced contractility (inotropy), faster heart rate (chronotropy) and faster relaxation (lusitropy), mainly through activation of protein kinase A (PKA). Efficient enhancement of heart output under stress requires all of these responses to work together. Lusitropy is essential for shortening the heartbeat when heart rate increases. It therefore follows that, if the lusitropic response is not present, heart function under stress will be compromised. Current literature suggests that lusitropy is primarily achieved due to PKA phosphorylation of troponin I (TnI) and phospholamban (PLB). It has been well documented that PKA-induced phosphorylation of TnI releases Ca^2+^ from troponin C faster and increases the rate of cardiac muscle relaxation, while phosphorylation of PLB increases SERCA activity, speeding up Ca^2+^ removal from the cytoplasm. In this review we consider the current scientific evidences for the connection between suppression of lusitropy and cardiac dysfunction in the context of mutations in phospholamban and thin filament proteins that are associated with cardiomyopathies. We will discuss what advances have been made into understanding the physiological mechanism of lusitropy due to TnI and PLB phosphorylation and its suppression by mutations and we will evaluate the evidence whether lack of lusitropy is sufficient to cause cardiomyopathy, and under what circumstances, and consider the range of pathologies associated with loss of lusitropy. Finally, we will discuss whether suppressed lusitropy due to mutations in thin filament proteins can be therapeutically restored.

## Introduction

It is 10 years since we wrote a review, based mainly on *in vitro* work, proposing that a major defect commonly connected with inherited cardiomyopathies (and perhaps some others) was their lack of response to TnI phosphorylation (1). The modulation of cardiac muscle relaxation rate due to TnI and phospholamban phosphorylation is a key determinant of the lusitropic response. This lead us to propose that suppression of lusitropy could be a disease mechanism in cardiomyopathies. This review revisits the question in the light of recent research.

Calcium ions (Ca^2+^), released from the sarcoplasmic reticulum, bind to troponin C to switch the thin filament on, however, in cardiac muscle a more graded form of regulation is essential to tailor cardiac output to the body’s needs. The level of contractility of heart muscle is determined by three factors: the initial sarcomere length (preload), the force against which the muscle must shorten (afterload), and the speed and force of contraction. Speed and force of contraction can be modulated independently of preload and afterload by changing the inotropic state. This is controlled largely, but not exclusively, by stimuli from the sympathoadrenal system and the parasympathetic nervous system.

Adrenergic activation acts mainly on β1 receptors to trigger a coordinated response of the heart during exercise or “flight-or-flight” that increases cardiac output up to fivefold. This is achieved by an increase in heart rate of up to threefold (chronotropy) and an increase in the force of contraction (inotropy). Ventricular pressure rises quicker and higher arterial pressure is produced. At the same time the duration of systole grows briefer and relaxation is faster (lusitropy).

The increased speed of relaxation is essential for the adrenergic response since the heart beat must become shorter if the heart rate is increased to avoid successive beats overlapping which would reduce stroke volume. Nevertheless, lusitropy is not often given the attention it deserves since heart rate and magnitude of contraction are obvious and easily measured parameters whilst relaxation rates are not commonly recorded. It is the objective of this review to consider the evidence that defects in lusitropy can be a significant contributor to heart disease.

The biochemical and physiological process of lusitropy is well understood. β-1 receptor activation leads to adenylate cyclase activation and cAMP production. cAMP acts directly on membrane channels and also activates the cyclic AMP-dependent protein kinase (PKA). PKA itself phosphorylates a variety of ion channels, ion pumps in the sarcolemma and sarcoplasmic reticulum (SR) and contractile proteins.

The SR in cardiac myocytes stores large quantities of Ca^2+^. The SR is a complex cellular compartment that allows intracellular Ca^2+^ cycling that is coordinated with other cellular systems such as myofilament and sarcolemma proteins. Ca^2+^ release from the SR is driven by the opening of Ryanodine Receptor whilst Ca^2+^ reuptake by the SR is mostly dictated by the Ca^2+^ pump called sarco/endoplasmic reticulum Ca2 + -ATPase (SERCA2a). In the sarcoplasmic reticulum PKA phosphorylates Phospholamban (PLB), an accessory protein crucial in the regulation of SERCA2a activity.

There are two phosphorylation sites in PLB, one at serine 16 and another at threonine 17 and the kinases involved are PKA for serine 16 and Ca^2+^/CaM kinase for threonine 17. Unphosphorylated PLB is an inhibitor of SERCA2a; PKA-mediated phosphorylation of phospholamban relieves the inhibition, thus activating SERCA2a, resulting in faster sequestration of Ca^2+^ (lusitropy) and increased filling of the sarcoplasmic reticulum that contributes to positive inotropy. In addition, positive lusitropism is achieved by a PKA-mediated phosphorylation of troponin I, exclusively at serines 22 and 23 (2, 3); phosphorylation of TnI at these two serine sites increases the rate of Ca^2+^-release from troponin C (4). To terminate these events when plasma concentrations of β-agonists fall, cyclic adenosine monophosphate (cAMP) is hydrolyzed by the cyclic nucleotide phosphodiesterases, and protein phosphatases hydrolyze the protein-bound phosphate. As will be discussed later, it is probable that both PLB and TnI phosphorylation are necessary for effective positive lusitropy. It is possible other targets of PKA, such as the L-type Ca^2+^ channel (5), may influence lusitropy, but comprehensive data is lacking. Recent publications have described the molecular mechanisms of troponin and phospholamban phosphorylation modulation of function (6, 7).

## Evidence that lusitropy is necessary from PLB loss of function models

The key studies of the role of phospholamban in cardiac muscle regulation are based on a PLB knockout (KO) mouse model (8). In unloaded assays (e.g., myocytes) the relaxation rate is completely unresponsive to isoprenaline (Iso) compared with a 30% increase in WT myocytes. However, in isometric contractions lusitropy is merely blunted: decrease in tau relax was 17% vs. 30–50% in non-transgenic littermates (NTG). PLB KO has additional actions, since it activated SERCA2a: tau is lower than NTG and there is a substantial inotropic effect. However, it can be concluded that PLB phosphorylation is a mechanism for lusitropy. Pathogenic, exonic variants have been identified in the PLB gene associated with DCM; to date, there are six known *PLN* mutations linked to dilated cardiomyopathy (p.R9C, R9L, R9H, R14del, R25C, L39X) (9–11) with several more candidate mutations suggested from large scale surveys [e.g., (12)].

The PLB R9C variant associated with DCM has been extensively studied for its inotropic and lusitropic effects in transgenic mice, cardiomyocytes and human iPSC-derived cardiomyocytes. Cardiomyocytes isolated from transgenic mice bearing the PLB R9C in the heart display prolonged Ca^2+^ transients. Upon further investigation it was found that the PLB R9C mutation “traps” PKA and inactivates it, preventing phosphorylation. This has an constitutive inotropic and lusitropic effect but yields negative consequences of impaired frequency potentiation and blunted β-adrenergic responsiveness (9). Another report suggested that acute expression of PLB R9C in cardiomyocytes enhances inotropic and lusitropic responses of the transfected cells but also blunts the response to the frequency of stimulation and isoproterenol stimulation (13). The PLB R9C mutation was also shown to cause a blunted β-agonist response in human iPSC-CMs in experiments performed using 3D human EHTs (14).

## Evidence that lusitropy is necessary from TnI phosphorylation sites serine 22/23 loss of function models

The role of TnI phosphorylation in modulating contractility has been studied in several transgenic mouse models either substituting the unphosphorylatable slow skeletal TnI for native cardiac TnI or by modifying the phosphorylatable serines, to non-phosphorylated (Ala substitution) or pseudophosphorylated (Asp or Glu substitution) forms.

**FIGURE 1:**
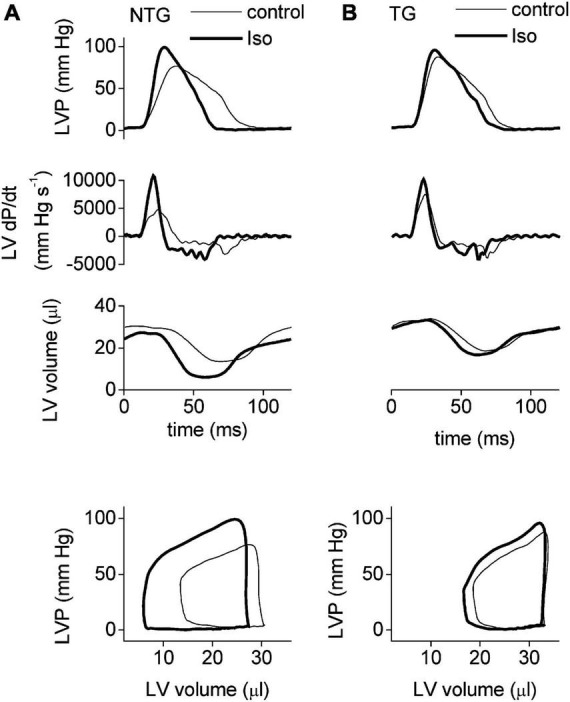
The adrenergic response in NTG **(A)** and ssTnI **(B)** TG mice. Examples of LV pressure traces (top set of panels), their derivatives (second set of panels), and the corresponding LV volume (third set of panels) recorded by a Millar microconductance catheter-manometer during ejecting heart experiments at 20 cm H_2_O preload. The bottom set of panels illustrate the steady-state pressure–volume loops derived from the instantaneous LV pressure and volume traces above (first and third panels). Thin lines indicate baseline conditions and thick lines indicate the signal during stimulation with isoprenaline (10 nm). Faster relaxation in presence of Iso is apparent in NTG but the effect is suppressed In the unphosphorylatable mutant TG mouse heart. Reproduced from Layland et al. (17) with permission.^©^ The Physiological Society 2004.

One of the most extensively studied models is the slow skeletal troponin overexpression model where cTnI in the heart is completely replaced with ssTnI that does not have the N-terminal phosphorylatable peptide (15–17). The substitution increases Ca^2+^ sensitivity of isometric tension in myofibrils (pCa_50_ is 5.81 WT, 6.13 TG) but also blunts the effect of PKA phosphorylation (Δ pCa_50_ = 0.146 in WT but 0.08 in TG). In myocytes, isoprenaline stimulation accelerated relaxation 1.7-fold in WT but only 1.3-fold in TG indicating a blunting of the lusitropic response that is also indicated by measurements of contraction kinetics on intact heart muscle strips (f_*min*_). In Langendorf working heart studies the effect of Iso on LVdp/dt_*min*_ is also blunted and the blunting of lusitropy is also evident in PV loops. The inotropic effect of Iso is not greatly altered in unloaded studies but, like the PLB KO, is partially blunted in loaded (auxotonic) muscles (Figure 1).

Substituting a non-native TnI and overexpression may have unforeseen physiological off target effects. Better models have been produced by mutating the phosphorylatable serines in native cTnI. The model described by Pi et al. overexpressed cTnI with ser22 and 23 mutated to Alanine (unphosphorylatable); moreover the transgenic mice were bred with cTnI null mice to avoid any interactions between native and transgenic cTnI (18, 19). The blunting of lusitropy was clear: as measured by ATPase in myofibrils or tension in skinned muscle fibers, the Ca^2+^-sensitivity was shifted 1.3-fold in NTG by Iso but no shift was detected in TG (18). In intact myocytes 2.5 nM Iso decreased relaxation time by 37% in WT but only 18% in TG. Yasuda et al. also studied Ser > Ala replacement in transfected myocytes, finding Iso decreased relaxation time by 36% in NTG but only 9% in TG (20).

The alternative model- substituting aspartic acid for the two serines to create a pseudophosphorylated troponin I has also been studied in transgenic mice and transfected myocytes. The first study used a mouse model overexpressing Asp 22/23 (21) and 95% replacement. It was noted that the mouse model was healthy but had enhanced cardiac function (as measured by Millar catheter) with faster contraction and slower relaxation, however, the response to β- adrenergic stimulation was not significantly different from NTG. Another group produced a similar model with either Asp 22/23 or Asp 22/23/43/45/144 overexpressed and studied lines with almost complete protein replacement (22). *In vitro* the Asp22/23 mouse myofibrillar Ca^2+^ sensitivity was lower than NTG, as expected for pseudophosphorylation (ΔpCa_50_ = 0.20), but upon PKA phosphorylation the Ca^2+^ sensitivity was not changed compared with ΔpCa_50_ of 0.23 in NTG. In the intact beating heart (Millar catheter), the rate of relaxation (dP/dT_*min*_) was increased from –6,869 to –8,651 mmHg/s by Iso in NTG and –10,598 to –12,099 in Asp22/23 mouse. Although the studies of Takimoto et al. and Sakthivel et al. show little evidence that loss of the TnI Ca^2+^-sensitivity shift causes lusitropy, the study by Yasuda et al. (20) using the same Asp22/23 model did show a blunting of lusitropy in myocytes. Myocyte twitch tension was measured by attaching microfibre probes. In Wild-type, ttb_75_ was decreased from 69 to 39 ms on treatment with Iso, a 44% change, whilst in Asp22/23 myocytes, ttb_75_ was decreased from 44 to 38 ms, a 14% change. A similar blunting was measured in myocytes transfected with Asp22/23: Iso produced a 33% reduction in relaxation time in WT but only 16% in Asp22/23. In addition, this study recapitulated the previous results with Ala22/23 and with ssTnI substitutions in both TG mouse and transfected myocytes (Table 1).

**TABLE 1:** Animal models of impaired lusitropy.

References	Model	Ca^2+^ sensitivity shift on phosphorylation, ΔpCa_50,_ WT > mutant	Lusitropy, % reduction of relaxation time, 100 × (1- relaxation time + dobu or Iso/relaxation time baseline), WT > mutant
(60)	PLB KO		30% > 0, isometric 40% > 17%
(15)	Ss TnI mouse	0.15 > 0.07	Unloaded 41% > 23%
(17)	ssTnI		dP/dT min 39% > 13%
(16)	Ss TnI	0.15 > 0.05	ttb_50_ skinned fibre 41% > 23%
(18)	cTnI Ser 23/24 Ala_2_	0.08 > 0	Unloaded 37% > 18%
(21)	cTnI 23,24 Asp_2_		
(22)	cTnI 23/24 Asp_2_	0.23 > 0.20	Loaded 26% > 14%
(20)	cTnI 23/24 Asp_2_ and Ala_2_		Asp_2_ isometric 44% > 14% Ala_2_ isometric 36% > 9%
(9)	PLB R9C		Ca^2+^ uptake rate, 20% > 0
(13)	PLB R9C		Unloaded, 29% > 24%
(14)	PLB R9C		Loaded EHT 25% > 4%
(61)	TnT I79N		26% > 9%, Langendorf 34% > 29%
(62)	TnT R278C		Langendorf 34% > 32%
(7)	TnT R92Q Guinea pig		Myocytes 24% > -9%
(63)	TnT R92Q	0.30 > - 0.04	Langendorf 45% > 13%
(53)	TnI P83S		Myofibrils: k_*RELslow*_ 56% > 9%
(55)	MYBPC3KI		IPSC eht 19% > 2%
(64)	TnTΔ160E		Millar catheter 28% > 19%
(44)	Actin E99K	IVMA 0.46 > 0.06 (mouse), –0.03 (human)	Echo, all Δ on dob suppressed
(65)	TnI R21C	0.25 > 0.05	32% > 5% (skinned fiber) 16% > 13% (myocytes)
(51)	TnI R21C		Exchanged myofibrils k_*RELslow*_ 39% > 8%, k_*RELfast*_ 14% > 5%
(59)	Actin E361G		Papillary muscle (10 Hz) 17.5% > 5% Millar catheter 22.4% > 7.5%
(66)	Actin E361G	Myofibrils 0.26 > -0.05	K_*RELfast*_ 34% > 0%
(37)	Actin E361G	IVMA 0.47 > 0.017	Cardiac output, dob effect Echo, 14% > 0%, MRI 19% > 8%

Where possible ttb_90_ is used. If not available, parameter is given in this table. All studies in mouse models unless otherwise stated. In paired *t*-test, wild-type mean lusitropy is 28.1% ± 1.8 (sem), mutant mean lusitropy is 11.0% ± 1.8 (sem), *n* = 25. t probability < 0.0001.

## “Is PLB or TnI the prima donna in β adrenergic induced lusitropy?”

This question was raised in a key Circulation Research editorial that discussed the findings of Yasuda in relation to the other published work at the time (23). It is still a very pertinent question. Essentially we need to know whether the release of Ca^2+^ from troponin or the removal of Ca^2+^ from the sarcoplasm by SERCA is rate limiting for relaxation. The answer to the question depends on the balance of rates of these two processes, which themselves depend on conditions, especially temperature since many experiments are conducted at below normal body temperature but also force, since these rates are different in unloaded and isometric muscle. Measurement technique, experimental medium and most likely also the animal model that is used are also critical.

Two studies have indicated that (in unloaded rat myofibrils at least) Ca^2+^ dissociating from TnC becomes rate limiting at 37°C whereas SERCA activity is limiting at lower, non-physiological temperatures (24, 25). Experiments do not support a dominating role for either PLB or TnI phosphorylation. Li et al. (8) proposed TnI phosphorylation contributes just 14–18% of lusitropy in unloaded muscle but nothing in isometric muscle based on comparison of WT with PLB-KO mouse. Yasuda et al. (20) predicted an opposite pattern with 75% contributed by TnI phosphorylation in unloaded myocytes. Layland et al. (17) argue that cTnI has the pivotal role in the positive inotropic response of the murine heart to β-adrenergic stimulation, under all loading conditions but agree that it is most evident in the auxotonically loaded ejecting heart.

It seems unlikely that either TnI or phospholamban phosphorylation is actually the “prima donna” but rather that the control of lusitropy is a duet. Wolska et al. (26) studied a transgenic mouse with both a PLB KO *and* ssTnI substitution. In isolated papillary muscle, the study confirmed that single mutant PLB KO enhances relaxation rate but uncouples it from Iso stimulation whilst single mutant ssTnI replacement slows relaxation and also uncouples. In the double mutant (PLBKO/ssTnI) relaxation is enhanced relative to wild-type (like the PLBKO) but the relaxation rate is still uncoupled from Iso stimulation. Thus, in the double KO TnI and PLB appear to act independently and additively. It is likely that phosphorylation of both PLB and cTnI contribute to the increased rate of relaxation during β-adrenergic stimulation and that deficiency of either will lead to suppression of lusitropy (23).

Somewhat disappointingly, none of these studies looked at the effects of chronic adrenergic stimulation. The transgenic mouse models often showed no cardiac phenotype at rest and this is entirely to be expected since sedentary mouse in the typical animal cage environment are not subjected to any stress. However, studies on mutations in troponin and other thin filament proteins that are demonstrated to be causative of dilated cardiomyopathy have provided a link between suppression of lusitropy and heart failure.

## Mutations can also suppress lusitropy

Many *in vitro* studies have demonstrated that mutations in thin filament proteins associated with inherited cardiomyopathies abolish the relationship between Ca^2+^ sensitivity and TnI phosphorylation by PKA and would therefore likely impair lusitropy. This trait, which we have termed “uncoupling,” was first noted in 2001 (27, 28). In 2007-8 three publications studying the recently discovered TNNC1 G159D mutation showed uncoupling with recombinant mutant troponin, with troponin extracted from a patient with the mutation and with rat trabecula with the mutation exchanged in myocytes (29–31). Subsequently, almost every thin filament mutation that was tested proved to be uncoupled. This was summarized by Messer and Marston (1) and Table 2 lists all the reports of uncoupling to date. In wild-type the mean ΔpCa_50_ is –0.31 (mean of 8 studies) (32) whereas it is close to zero in the mutations studied. It is relevant to note that in *in vitro* experiments, uncoupling is always complete, irrespective of the mutation and that uncoupling is associated with both HCM and DCM-causing mutations. In the case of DCM mutations a case can be made that this is causative of the disease since it is the only mutation-related property that is common to every thin-filament related DCM mutation (1, 33, 38), whereas for HCM, the increase in Ca^2+^-sensitivity is probably the key driver of the cardiomyopathy. Mechanistically, uncoupling usually involves an impaired response of the thin filaments to TnI phosphorylation (34), however, the cTnI R21C mutation has a mechanism whereby the mutation interferes with the phosphorylation process itself (35). Another mechanism that could generate impaired lusitropy is if the balance of phosphorylation of TnI and PLB in response to Iso is disturbed as suggested by Najafi et al. (36).

**TABLE 2:** Mutations that have been reported to cause uncoupling.

Mutation	Effect of phosphorylation on Ca^2+^-sensitivity, pCa_50_ uP- pCa_50_ P	Measurement method	Publication
***DCM***			
ACTC E361G	0.017	*In vitro* motility assay (IVMA)	(37, 38)
TPM1 E54K	0.021	IVMA	(38)
TPM1 E40K	0.00	IVMA	(38)
TPM1 D230N	-0.013	IVMA	(38)
TNNC1 G159D	-0.013	IVMA/Ca^2+^ binding	(29, 30, 38, 39)
TNNC1 Y5H	0.068	Skinned fiber	(40)
TNNC1 M103I	0.017	Skinned fiber	(40)
TNNC1 I148V	0.053	Skinned fiber	(40)
TNNT2 ΔK210	-0.009	IVMA/skinned fiber	(38, 41, 42)
TNNT2 R141W	-0.022	IVMA	(38)
TNNI3 K36Q	-0.009	IVMA/ATPase	(38, 43)
***HCM***			
ACTC E99K	-0.004	IVMA	(44, 45)
TPM1 E180G	-0.009	Skinned fiber	(45, 46)
TNNC1 L29Q	-0.036	Ca^2+^ binding/ATPase	(30, 47, 48)
TNNT2 R92Q	0.041	IVMA	(49)
TNNT2 Δ14	0.000	IVMA	(49)
TNNT2 Δ28 + 7	0.000	IVMA	(49)
TNNT2 ΔE160Q	0.000	IVMA	(49)
TNNT2 S179F	0.041	IVMA	(49)
TNNT2 K273E	0.041	IVMA	(49)
TNNT2K280N	0.041	IVMA	(49, 50)
TNNI3 R145G	0.000	IVMA/ATPase	(27, 51)
TNNI3 R145W	0.045	IVMA/ATPase	(52)
TNNI3 P83S	0.000	Exchanged myofibrils	(53)
TNNI3 R21C	0.049	Skinned fiber	(35, 51)
TNNI3 G203S	0.057	ATPase/IVMA	(28)
TNNI3 K206Q	0.000	ATPase/IVMA	(28)

There are a few reported cases of uncoupling were the mutation is in thick filament or cytoskeletal proteins or occurs with no known mutation (50, 54, 55), however, there is also clear evidence that DCM-associated titin truncation (TTNtv) mutations are fully coupled and that abnormal stiffness and disabled length-dependent activation may be more important for the pathology (56, 57).

**FIGURE 2:**
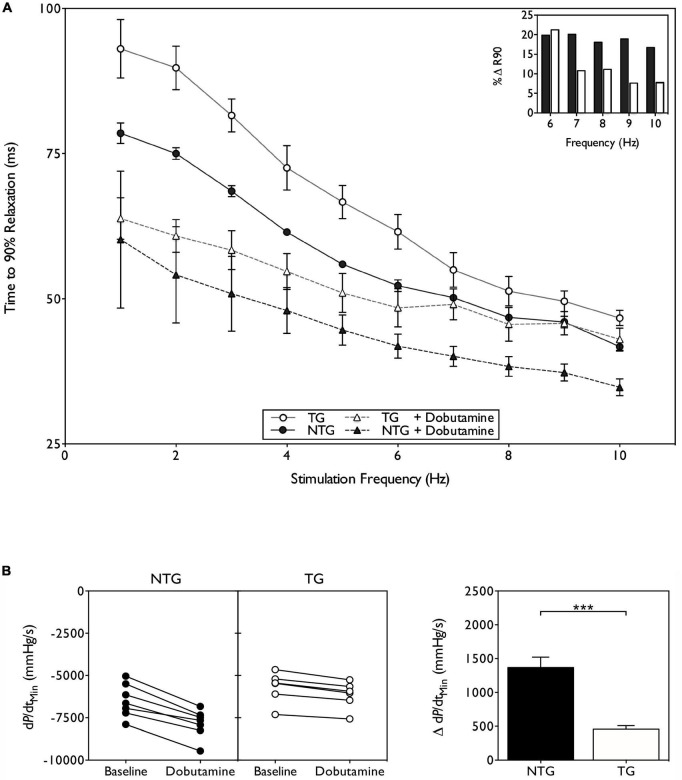
Suppression of lusitropy in ACTC E361G transgenic mouse. **(A)** Effect of dobutamine on intact papillary muscle contractility. Papillary muscles, isolated from both ACTC E361G (*n* = 5) and NTG mice (*n* = 5), were stimulated with the addition of 10 μM dobutamine to the perfusion solution at 37°C. Time to 90% relaxation is plotted against stimulation frequency; (inset) percent change of ttb_90_ with dobutamine treatment at physiologically relevant frequencies. **(B)** The effect of acute dobutamine treatment on cardiac performance determined by Millar catheter. Data are presented as mean ± SE of 6 ACTC E361G (open bars) mice and 7 NTG (solid bars) mice. The change of rate of pressure decline (dP/dtmin) on treatment with dobutamine is plotted (left) and mean values plotted in a bar chart (right). ***P = 0.001, unpaired student’s *t*-test. Data from Wilkinson et al. (59).

Uncoupling implies impaired lusitropy but this has only been tested in a few instances, listed in Table 1. Methodology varies widely: myofibril, iPSC contractility and myocyte contractility, trabecula and papillary muscles and Langendorf-mounted heart. In intact animal, Millar catheter, Echocardiography and Cine-MRI have been used. It is remarkable that mostly these techniques give a uniform result: a 30–50% reduction in ttb_90_ on adding PKA or β1 adrenergic stimulation in the WT compared to a 10–20% reduction in the HCM or DCM mutant animal. Figure 2 shows an example from the work of Wilkinson et al. A possible exception is the DCM-causing D230N mutations in tropomyosin (58) which is uncoupled in single filament assays (Table 2) but shows very little blunting of lusitropy in unloaded myocytes. In general, the range of the lusitropy parameter in cardiomyopathic mutant studies is very similar to the phosphomimetic transgenic animals, confirming that uncoupling due to mutations suppresses lusitropy (59) and revealing this important role for cTnI phosphorylation in cardiac muscle regulation.

The next stage is to determine whether impaired lusitropy can lead to cardiac dysfunction. The transgenic mice studies have found that the majority of DCM mutations in thin filament proteins produce little or no cardiac phenotype, with the exception of a few TnT and Tm mouse models (41, 67, 68). This is to be expected if the defect is in the response to stress.

Very few studies have considered whether chronic stress could induce cardiac dysfunction. The most well documented study is by Wilkinson et al. (59). This study used the ACTC E361G DCM-associated mouse model with the mutant expressed at 50% of total actin, as was commonly found in patients. Initial studies indicated that the loss of lusitropy was the only cardiac dysfunction linked to the mutation and that the mice had a normal phenotype at rest up to 22 months (37). It was hypothesized that the loss of cardiac reserve due to suppressed lusitropy would predispose the ACT E361G mouse hearts to failure under chronic adrenergic stress. Mice were treated with high doses of Angiotensin II applied by osmotic minipump for 4 weeks. In E361G mice the angiotensin II treatment induced mild systolic dysfunction, as measured by Millar catheter, whilst having no effect on the NTG controls. Compared with the NTG mice ACTC E361G mice had significantly lower rates of pressure increase and decrease as well as reduced end-systolic pressure. As a result cardiac output and ejection fraction were approximately half the value in NTG, indicating that uncoupling had induced contractile dysfunction under chronic stress, characteristic of the early stages of DCM.

## Restoration of lusitropy as a potential treatment for cardiomyopathies

Studies on the effects of mutations that uncouple TnI phosphorylation from the Ca^2+^-sensitivity shift indicate that they probably act by inducing a subtle, phosphorylation-dependent change in the dynamics of troponin (69–74), most clearly indicated by a recent study on the TnC G159D mutation (34). Remarkably, it has been found that several small molecules are capable of fully restoring the Ca^2+^-sensitivity shift *in vitro* and restoring lusitropy to mutated cardiomyocytes (7, 45, 75, 76). These small molecules (EGCG, SilybinB, Resveratrol) therefore have potential for treatment of cardiac dysfunction due to suppression of lusitropy, particularly by thin filament mutations but also as a tool for probing for cardiac dysfunction due to suppression of lusitropy. For instance, Tadano et al. in HCM mouse (77) and Mou et al. (78), using a rat abdominal aortic constriction model of heart failure, have demonstrated that EGCG corrects cardiac systolic and diastolic dysfunction and prevents cardiac remodeling.

## Is there a connection between suppression of lusitropy and human cardiomyopathy?

Based on the animal studies of the consequences of uncoupling, we have proposed that patients with uncoupling mutations that are associated with DCM would have impaired lusitropy and that this could contribute to the heart failure phenotype. A thorough investigation of the state of knowledge of idiopathic DCM around this possibility has revealed no direct evidence for the possibility since the question has not been investigated.

There is indirect evidence compatible with our hypothesis, although, of course, other mechanisms may play a part. Mutations that cause suppressed lusitropy also have a reduced contractile reserve when challenged with β1 agonists in transgenic mouse models (59, 79). A significant association between the absence of left ventricular contractile reserve and increased rate of cardiovascular events, cardiac death and all- cause mortality has been demonstrated (80). Several studies have reported that IDCM patient cohorts tend to segregate in to two groups: those that respond normally to dobutamine and those with a reduced response, usually measured as contractile reserve (Table 3). Moreover, the patients with a reduced response to dobutamine have a more severe prognosis that the other group and also do not respond to standard heart failure medication [see meta-analysis by Waddingham et al. (81)].

**TABLE 3:** Studies showing that reduced response to adrenergic stimulation is correlated with cardiac adverse outcome in patients with idiopathic DCM.

Paper	Non-responders, responders	Outcome measured	Follow up time	Adverse events (responders)	Adverse events (non-responders)
(84)	38, 33	Cardiac mortality	60 mo	9%	42%
(85)	11, 10	Δ LVEF, Δ LV sphericity	6 mo	0%	45%
(86)	11, 7	Δ LVEF improvement	15 mo	0%	36%
(87)	83, 103	Cardiac mortality	15 mo	3%	25%
(88)	89, 43	Mortality and/or hospitalization	40 mo	16%	49%
(89)	13, 24	Cardiac mortality	60 mo	34%	76%
(90)	15, 28	Mortality and/or hospitalization	23–67 mo	11%	67%

Data partially based on Waddington (81).

The behavior of the non-responders matches that which would be expected if they had familial DCM mutations that suppressed lusitropy whereas the responders probably correspond to patients with idiopathic DCM caused by non-genetic factors where, both at the single filament and patient levels, lusitropy has been demonstrated to be normal (82, 83).

Unfortunately this hypothesis has not been tested in IDCM patients. There is no clinical study that correlates thin filament mutations with reduced response to β1 stimulation, nor is lusitropy (relaxation rate increase or twitch duration decrease) commonly measured in Echo or MRI analysis of patients although protocols to do so are available. To test this hypothesis we need to know whether cases of familial DCM caused by mutations correlate with the non-responders group and also whether lusitropy is suppressed in the non-responders.

It is possible that suppression of lusitropy could also play a role in other cardiomyopathies. It is consistently observed that thin filament mutations that cause HCM are associated with uncoupling (Table 2) and loss of lusitropy in cells and intact animals (see ACTC E99K, TNNT2 R92Q in Table 1). Enhanced Ca^2+^-sensitivity is the predominant effect of HCM mutations (91–95). Impaired relaxation is characteristic of HCM. Suppression of lusitropy may play a role here, however, it would be very difficult to unravel what contribution loss of lusitropy makes to the HCM phenotype since the increased Ca^2+^ sensitivity and the suppressed lusitropy would both compromise relaxation.

HFpEF is a heterogeneous disease associated with diastolic dysfunction. HFpEF patients have a high prevalence of a blunted response to exercise which may be linked to suppressed lusitropy. However, this has not been measured in any of the studies to date (96, 97).

## Conclusion

Basic and animal studies indicate that specific suppression of lusitropy can induce symptoms of heart failure under stress. In the human heart, where stress is far more common than in laboratory animals, there is considerable circumstantial evidence that impaired lusitropy whether due to cardiomyopathic mutation or other causes, can contribute to heart failure. It is predicted that such symptoms of heart failure would not be amenable to conventional heart failure therapy but may benefit from small molecules that have been shown to restore lusitropy in laboratory studies. We believe these issues should be taken into account in clinical investigations of cardiomyopathy.

## Author contributions

Both authors listed have made a substantial, direct, and intellectual contribution to the work, and approved it for publication.
